# Characterization of Leaf Transcriptome in *Banksia hookeriana*

**DOI:** 10.1016/j.gpb.2016.11.001

**Published:** 2017-02-02

**Authors:** Sim Lin Lim, Haylee M. D'Agui, Neal J. Enright, Tianhua He

**Affiliations:** 1 Department of Environment and Agriculture, Curtin University, Perth, WA 6845, Australia; 2 School of Veterinary and Life Sciences, Murdoch University, Perth, WA 6150, Australia

**Keywords:** *Banksia hookeriana*, Gene annotation, RNA-seq, SSR marker, Transcriptome

## Abstract

*Banksia* is a significant element in vegetation of southwestern Australia, a biodiversity hotspot with global significance. In particular, ***Banksia hookeriana*** represents a species with significant economic and ecological importance in the region. For better conservation and management, we reported an overview of **transcriptome** of *B. hookeriana* using **RNA-seq** and *de novo* assembly. We have generated a total of 202.7 million reads (18.91 billion of nucleotides) from four leaf samples in four plants of *B. hookeriana*, and assembled 59,063 unigenes (average size = 1098 bp) through *de novo***transcriptome** assembly. Among them, 39,686 unigenes were annotated against the Swiss-Prot, Clusters of Orthologous Groups (COG), and NCBI non-redundant (NR) protein databases. We showed that there was approximately one single nucleotide polymorphism (SNP) per 5.6–7.1 kb in the **transcriptome**, and the ratio of transitional to transversional polymorphisms was approximately 1.82. We compared unigenes of *B. hookeriana* to those of *Arabidopsis thaliana* and *Nelumbo nucifera* through sequence homology, Gene Ontology (GO) annotation, and KEGG pathway analyses. The comparative analysis revealed that unigenes of *B. hookeriana* were closely related to those of *N. nucifera*. *B. hookeriana*, *N. nucifera*, and *A. thaliana* shared similar GO annotations but different distributions in KEGG pathways, indicating that *B. hookeriana* has adapted to dry-Mediterranean type shrublands via regulating expression of specific genes. In total 1927 potential simple sequence repeat (SSR) markers were discovered, which could be used in the genotype and genetic diversity studies of the *Banksia* genus. Our results provide valuable sequence resource for further study in *Banksia*.

## Introduction

*Banksia* (Proteaceae) consists of 173 recognized species that are endemic to Australia except one species, *B. dentata*, which extends to New Guinea and Aru Island [1], [2]. This genus is a significant taxa group in southwestern Australia where a high human population density coincides with a highly-fragmented landscape to increasingly threaten species persistence [1]. *Banksias* species range from prostrate shrubs to trees up to 30 m [3], which have developed extraordinary adaptations to recurrent fires [4]. *B. hookeriana* is shrub with narrow distribution in fire-prone vegetation of the northern Sandplains in southwestern Australia [5]. It was for many years the most important species for the wildflower industrial in Australia [6]. The combination of commercial wildflower harvesting, altered fire regime, and vegetation clearing for farm and mining has led to the species’ range to be reduced by ∼40% in area since 1960 [7]. Moreover, this species has been shown to be sensitive to climate change, particularly drought [5].

Despite its importance to studies of evolution and conservation in fire-prone environments [8], [9], the genomic resources available for the study of *Banksia* are limited. Currently, there are only 1091 *Banksia* DNA sequences deposited in public database such as the NCBI database (released in December 2016). Most of these *Banksia* sequences have been only used for phylogenetic and diversification studies [10]. Since the number of genes in *Banksia* is unknown, characterization and annotation of genes from transcriptome is essential. RNA-seq, also termed as “whole transcriptome shotgun sequencing”, is often used nowadays to analyze species transcriptomes [11], [12]. RNA-seq can generate millions of short cDNA reads [13], which are subsequently aligned to a reference genome or *de novo* assembled, providing significant information about transcriptional structure and gene expression pattern without sequencing the whole genome. Using RNA-seq, transcriptomes of *Hevea brasiliensis*, *Trifolium pratense*, *Agave deserti*, and *Agave tequilana* had been assembled *de novo* recently [14], [15].

In the present study, we used *B. hookeriana* as a representative of the *Banksia* genus for RNA-seq analysis. We generated over 18.91 billion nucleotides of DNA sequences with high quality for gene assembly and annotation in species without prior available genomic information. The Gene Ontology (GO) annotation and KEGG pathway analysis for *B. hookeriana* unigenes were also performed in comparison with closely-related species with transcriptome data available, including *Nelumbo nucifera* and the model organism *Arabidopsis thaliana*. We further investigated the heterozygosity and genetic variability between different samples, and developed a large number of simple sequence repeat (SSR) markers that are associated with expressed genes. These results provide discovery of new ecologically-related functional genes, novel single nucleotide polymorphisms (SNPs), and potential SSR markers in the *Banksia* genus.

## Results

### *De novo* assembly of *B. hookeriana* transcriptome

**Supplementary Figure S1:**
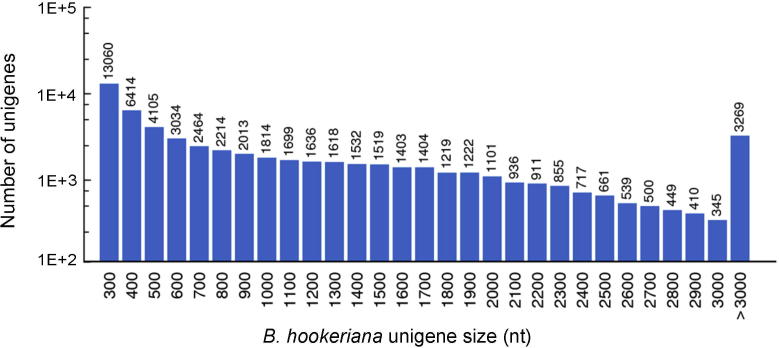
Length distribution of *B. hookeriana* unigenes

**Figure 1:**
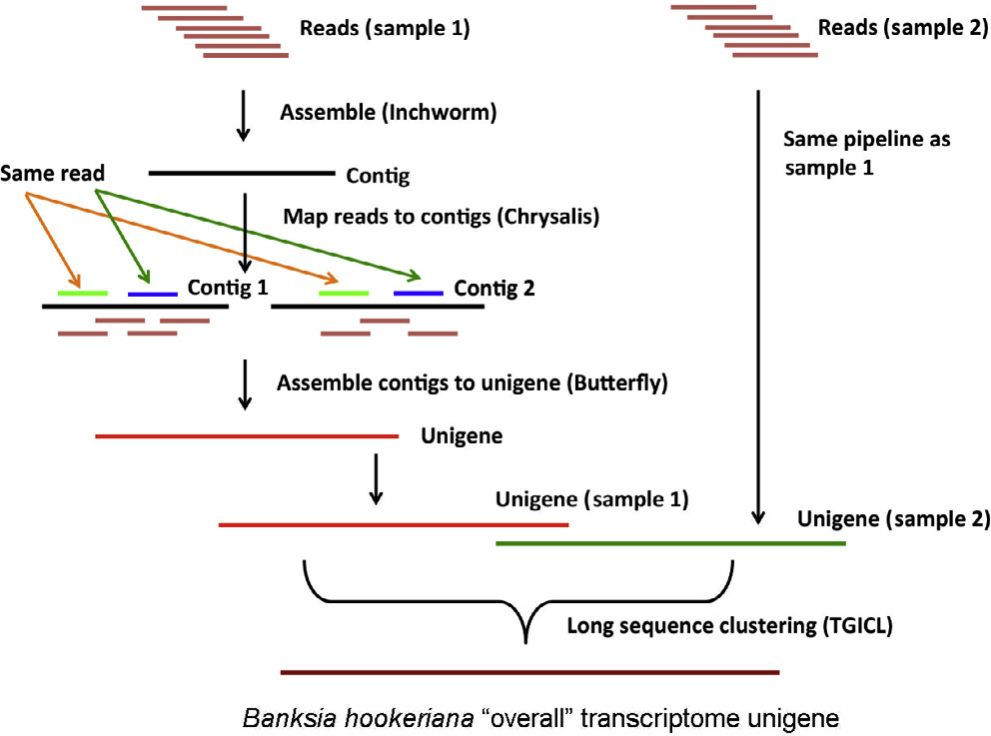
*De novo* assembly pipeline of *Banksia hookeriana* leaf transcriptome

Transcriptome analysis was performed for four fresh leaf samples from four plants of different *B. hookeriana* populations by RNA-seq (Figure 1). On average, 47,287,067 clean reads were generated (Table 1). Among them, there were 46,289,310 (97.89%) high-quality reads (Q > 20) and no reads contained “N” (Table 1). An average of 99,304 contigs was assembled from these high-quality reads (Table 2). The length of contigs ranges from 100 nt to 12,556 nt with an average of 402 nt. An “overall” assembly for *B. hookeriana* was generated, which contained 59,063 unigenes (Table 2). Among them, 25,912 unigenes are distinct clusters, and 33,151 unigenes are distinct singletons in the “overall” assembly. The average size of the “overall” assembly unigenes was 1098 nt (Table 2), ranging from 300 nt to ≥3000 nt (Figure S1).

### Functional annotation of unigenes

For functional annotation, the *B. hookeriana* “overall” unigenes were searched against three databases using BLASTX. Out of 59,063 unigenes, 27,462 unigenes (46.03%) were annotated to proteins in Swiss-Prot, 12,147 in NCBI NR, and 77 in Clusters of Orthologous Groups (COG) databases, respectively (available in Dryad repository doi:10.5061/dryad.60vj4). The remaining 19,377 genes were identified with unknown functions. Further analysis revealed that only 13 sequences were aligned with tRNA or rRNA sequences (available in Dryad repository doi:10.5061/dryad.60vj4). No transposable elements were annotated in these unigenes. A total of 22,194 (37.5%) unigenes are in the 5′–3′ direction. The presence of full-length assembled unigenes was detected and we found that 11,505 unigenes matched proteins in the Swiss-Prot database by 80%−100% of their protein lengths (Table S1). Among the 39,686 annotated unigenes, 52.6% (20,864) matched with proteins from *Vitis vinifera* and 9.3% (3695) with *Amygdalus persica*, whereas 38.1% (15,127) were directly aligned to other species proteins.

### SNPs in expressed genes

**Supplementary Figure S2:**
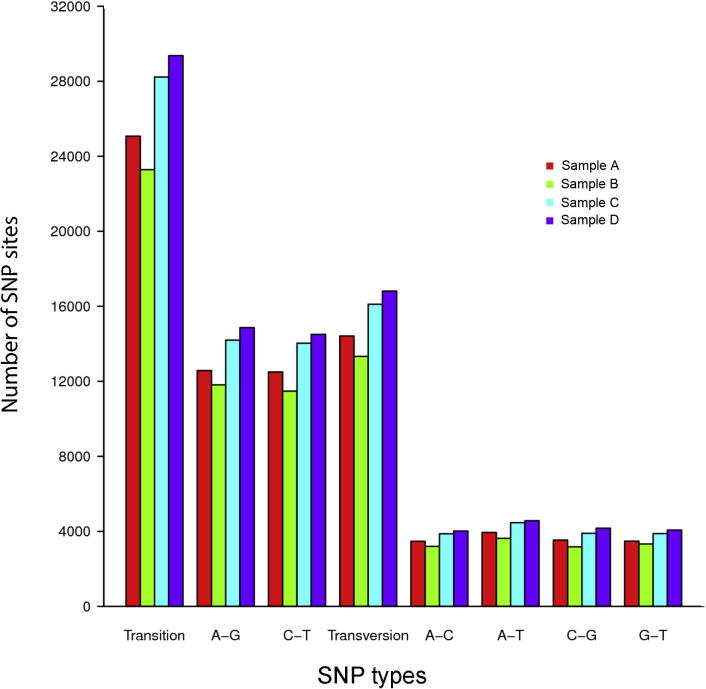
SNP variations found in different *B. hookeriana* transcriptomic samples

Nucleotide sequence of the assembled 59,063 unigenes was used for SNP discovery. Sequencing reads from the *B. hookeriana* samples were mapped back to the reference to call SNP. A total of 105,597 SNPs was found in 24,490 reference unigenes (available in Dryad repository doi:10.5061/dryad.60vj4). The majority of the SNPs discovered in samples A (72.0%), C (72.0%), and D (72.3%) were polymorphic, whereas sample B (44.9%) showed the lowest heterozygosity and polymorphism (Table 3). The SNP calling allowed us to discover approximately 1 SNP per 5.6–7.1 kb. Further analysis revealed that the majority (∼63.59%) of the SNPs found in *B. hookeriana* samples were transition, while the remaining SNPs were transversion (∼36.41%) (Figure S2; available in Dryad repository doi:10.5061/dryad.60vj4).

### Comparison of transcriptomes among *B. hookeriana*, *N. nucifera*, and *A. thaliana*

*B. hookeriana* is closely-related with *N. nucifera*, whose transcriptome had been reported [16]. To better understand the organization of transcriptome in a phylogenetic context*,* we further compared for similarity and difference of the *B. hookeriana* overall transcriptome with those of *N. nucifera* and the model plant *A. thaliana* [17] using TBLASTX (http://nebc.nox.ac.uk/bioinformatics/docs/tblastx.html). We found that 2984 unigenes of *B. hookeriana* had a one-to-one relationship with those from *N. nucifera*, whereas 32,739 unigenes of *B. hookeriana* could be mapped to multiple transcripts of *N. nucifera* (available in Dryad repository doi:10.5061/dryad.60vj4). Similar observation was made for the comparative analysis of *B. hookeriana* and *A. thaliana* unigenes, with one-to-one relationship found for 2462 unigenes and mapping to multiple transcripts for 32,068 unigenes of *B. hookeriana* with those of *A. thaliana* (available in Dryad repository doi:10.5061/dryad.60vj4). The TBLASTX analysis showed that approximately 23,340 *B. hookeriana* unigenes were not related to those of *N. nucifera* and *A. thaliana*.

**Figure 2:**
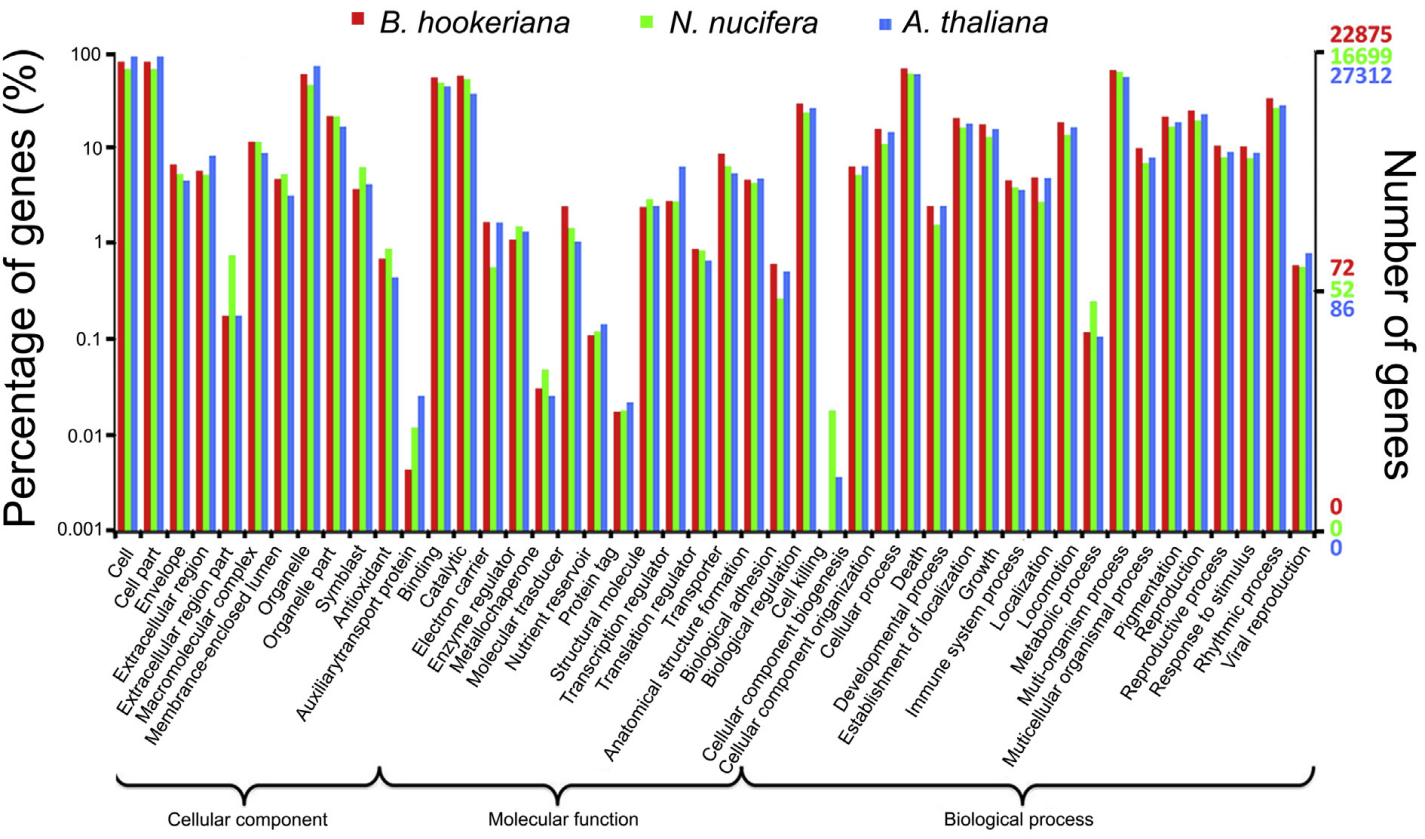
GO annotation analysis of *B. hookeriana*, *N. nucifera*, and *A. thaliana* transcriptomes

We annotated the 59,063 unigenes with GO [18] classifications. Based on sequence homology, 22,875 (38.7%) of *B. hookeriana* unigenes were categorized into 51 functional second-level GO term groups (Figure 2). A high percentage of genes were categorized into “cell”, “cell part”, “cellular process”, “organelle”, and “metabolic process”. The comparison of *B. hookeriana* with *N. nucifera* and *A. thaliana* transcriptomes revealed that the percentage distributions of GO annotations in these transcriptomes are similar, and 295 out of 470 (62.8%) third-level GO terms were significantly enriched (*P* < 0.05) within three species (Table S2).

We then performed pathway analysis using a BLASTX search in the KEGG database [19] and found that 26,724 (45.2%) *B. hookeriana* unigenes are assigned to 128 pathways. The most enriched pathway groups included metabolic pathways (13.86%), biosynthesis of secondary metabolites (8.36%), plant–pathogen interactions (5.31%), plant hormone signal transduction (3.88%), and spliceosome (2.49%) as shown in Table 4. However, the KEGG annotations of the other two species showed slightly different distributions. The four most enriched pathway groups for *N. nucifera* and *A. thaliana* were plant hormone signal transduction, biosynthesis of secondary metabolites, metabolic pathways, and protein processing in endoplasmic reticulum and ribosome.

### Mining SSR markers from unigenes

To obtain SSRs for population genetics analysis, we have identified a total of 9887 potential SSR markers from the RNA-seq of four *B. hookeriana* samples. There were five SSR types, including di-SSR (629), tri-SSR (1075), tetra-SSR (45), penta-SSR (55), and hexa-SSR (123) (Table S3). The penta-SSRs accounted for 34.04% (656) of all SSR identified (Table S3).The average repeat number in those SSRs was 6.1, ranging from 4 to 13.

## Discussion

The advancement of high-throughput sequencing technologies and decreasing sequencing cost are making changes in current research settings. RNA-seq technology provides ultra high-throughput sequencing data at an affordable cost, and has opened a door for new analyses in multiple research fields including population genetics and ecological adaptation [20]. In this study, we successfully sequenced transcriptomes of 4 *B. hookeriana* samples from different populations. We generated 18.91 billion nucleotides screened from 46.62 Gb of raw sequence data by Illumina Hiseq2000 sequencing. We assembled these reads into 59,063 unigenes with the mean size of 1098 bp (Table 2), which is similar to other *de novo* transcriptome assembly (712–1132 bp) by using Trinity [21], [22]. Among 27,462 unigenes that were successfully aligned with known proteins in the Swiss-Prot database, 859 unigenes were either classified as “hypothetical” or “putative” proteins. However, the majority (79.5%) of the assembled unigenes were assigned to NCBI NR database with unknown protein properties, which made functional prediction of those genes difficult.

SNPs can be readily characterized using genomic or transcriptomic sequences [23]. By using 59,063 unigenes from the assembled transcripts as references, we generated a database that contains 105,597 SNPs. Our data showed that approximately half of the predicted genes contain at least one SNP site. Transition/transversion ratio of SNPs in remained similar across the four *B. hookeriana* leaf samples examined (∼1.82), which is slightly higher than eggplant (1.65), rubber tree (1.67), sunflower (1.72), and oil palm (1.78) [24], [25], [26], [27]. The bias in transition/transversion ratios in SNP discovery could be explained: (1) possible selective pressure for gene conservation [28]; (2) transitions observed more often than transversions in synonymous substitutions [29]; and (3) frequent deamination of 5-methylcytosine to thymine [30]. Our SNP database would facilitate further analysis of gene expression, mutation, and polymorphic evolution for *B. hookeriana* in the future.

Functional annotation, classification, and comparative analysis provide useful information on metabolic pathways of *B. hookeriana*. Different annotation procedures could provide a range of details and insights into gene function. Both classifications promote the understanding of *B. hookeriana* gene functions and predicting the unigene’s potential physiological roles. The TBLASTX result revealed that the *B. hookeriana* unigenes are more closely related to the *N. nucifera* than to the *A. thaliana* transcript. This is expected because *N. nucifera* and *B. hookeriana* belong to the same order (Proteales) and share a common ancestor around Creataceous period [∼100 million years ago (mya)], while *A. thaliana* is a more distantly related species (Brassicales) that appeared around Neogene period (∼23 mya) [31], [32]. Proteales and Brassicales only share common ancestor in Jurassic period (>150 mya). The GO classifications revealed similar transcript distribution in biological processes, cellular components and molecular functions for these three species, whereas the KEGG annotation suggested that *B. hookeriana* genes are enriched in slightly different metabolic pathways compared with the other two species. We speculate that environmental factors affect the metabolic pathways. For example, *A. thaliana* grows in agricultural fields, disturbed sites or forest openings [33] and *N. nucifera* grows in tropical area where they could obtain the soil nutrients easily [34]. However, *B. hookeriana* was only found in dry-Mediterranean type shrublands that contains low soil nutrients and moisture [5]. Such condition could induce *B. hookeriana* adaptation to abiotic stress via up-regulating or down-regulating specific gene expression in the leaf that could contribute to alternate metabolic pathways. Further experiments are required to validate the differential KEGG pathways observed across these three species and investigate the KEGG pathway connections with plant environmental adaptations.

As demonstrated in our study, transcriptome sequencing of non-model species provides a significant amount of DNA sequences for SSR markers development. He et al. [35] developed 11 polymorphic SSR markers for *B. hookeriana* using a magnetic bead-based enrichment procedure [36], but the markers were limited in usage without genomic or transcriptomic assembly reference. We performed an extensive screening of *B. hookeriana* transcripts to search potential SSR markers in these unigenes. The resulting extensive list of SSRs would have significant implication in population and conservation genetics, comparative genomics, and identification of quantitative trait loci [37]. Given these potential SSR markers were identified only based on computational analysis, further experimental work is required to validate them.

In conclusion, our RNA-seq analysis and *de novo* assembly provide the first overall view of the non-model plant *B. hookeriana* transcriptome. The RNA-seq analysis also provides the first insights of *B. hookeriana* gene functional annotations and the discovery of potential SSR markers that have not been reported before. Our study provides comprehensive genomic information for further research into the functional ecology and conservation management of *B. hookeriana*.

## Material and methods

### Plant material and RNA isolation

Seeds of *B. hookeriana* were collected from four populations near Eneabba, Western Australia where *B. hookeriana* is narrowly distributed and populations are connected genetically through pollen flow and seed dispersal [5]. Seeds were extracted from fruits and germinated in Petra-dish with wet filter paper at 15 °C. Germinant seedlings were sown into 100 cm × 15 cm of tube pots containing low nutrient acid sands (Bassendean sand) [38]. Seedlings were grown in a greenhouse where they were watered every two days for 10 weeks before being sampled. Leaf samples of one plant from each population were selected for RNA isolation. Leaves were immediately cleaned with diethypyrocarbonate-H_2_O, and stored in RNAlater (Life Technologies Australia, Mulgrave, Australia). The samples were frozen with liquid nitrogen and blended into fine powders.

### RNA sequencing

Total RNA was isolated using the Trizol method [39] and mRNAs were then isolated using beads with oligo (dT), before being fragmented in fragmentation buffer. cDNAs were synthesized using these short fragments as templates and a random hexamer as a primer, and then purified using QiaQuick PCR Purification Kit (QIAGEN, Duesseldorf, Germany). The purified short fragments were dissolved in elution buffer for end reparation and single nucleotide A (adenine) addition. cDNAs were added to adapters, and fragments with length ∼160 bp were selected for the PCR amplification. Agilent 2100 Bioanaylzer (Agilent Technologies, Palo Alto, CA) was used in quantification and qualification of the sample library, and qPCR was used to detect library concentration. Finally, the library was sequenced using Illumina HiSeq2000 (Illumina, San Diego, CA) at Beijing Genomics Institute (BGI, Shenzhen, China). The Illumina Hiseq2000 was set with 100 cycles for the pair-end sequencing. Software “filter_fq” was used to determine the quality of reads. The quality read was determined by sQ = −10lgE, where sQ represents the sequencing quality value and E represents sequencing error rate. If the rate of reads with sQ value ≤10 was more than 20%, they were considered as low quality and removed. “filter_fq” was also used to screen for potential short-read contaminations. Reads with unknown nucleotides >5% were removed. We defined the reads with sQ > 20 and no ambiguous sequences “N” as high-quality reads.

### Transcriptome *de novo* assembly

*De novo* assembly of transcriptome was performed using a de Bruijn graph and Trinity [40] that consists of three independent programs: Inchworm, Chrysalis, and Butterfly [41] (Figure 1). Inchworm firstly assembled the RNA-seq data into the unique sequences of transcripts (contigs) with a defined overlap length (k-mer = 25) and minimum overlap coverage of three reads. The resulting contigs were then clustered by Chrysalis into clusters. In the final step, the individual graphs were processed in parallel using Butterfly, and full-length transcripts for alternatively-spliced isoforms (unigenes) for each sample (A, B, C, and D) were reported. The Trinity software settings were based on recommendation of Grabherr and colleagues [40], as the *B. hookeriana* genome is not yet available. When four samples of *B. hookeriana* were sequenced and assembled, unigenes from assembly of each sample were clustered by TGICL software to assemble “overall” transcriptome assembly that contained non-redundant unigenes as long as possible [42]. The gene family clustering was done on the leaf samples and “overall” transcriptome unigenes, which were divided into two categories: (1) cluster type, where sequence similarity between several unigenes is more than 70%, and (2) singletons type, where the unigene did not show any similarity with other unigenes.

### Annotation and classification of unigenes

Unigene sequences were first aligned by BLASTX (https://blast.ncbi.nlm.nih.gov/Blast.cgi) to protein databases including COG (http://clovr.org/docs/clusters-of-orthologous-groups-cogs/), Swiss-Prot (https://www.ebi.ac.uk/uniprot), and KEGG (http://www.genome.jp/kegg) using a cut-off E-value of 1E–5 [43], [44]. These sequences were further used for BLASTX searches and annotation against an NCBI NR protein database (https://www.ncbi.nlm.nih.gov/protein) using a cut-off E-value of 1E–10 [45]. If a protein showed highest similarity in DNA sequence with a given unigene, the protein information and functional annotations were retrieved. When there is disagreement in results from different databases, we followed a priority order of Swiss-Prot, NCBI NR, COG, and KEGG. If not mapped to any known database, the unigene was then aligned by BLASTN to tRNA and rRNA database with a cut-off E-value of 1E–5. RepeatMasker (http://www.repeatmasker.org) was used to search any potential transposable elements [46]. The presence of full-length assembled unigenes was detected by using the Perl script analyze_blastPlus_topHit_coverage.pl provided in Trinity [47], [48].

### SNP discovery

The unigenes assembled from Trinity was used as the reference genome for SNP discovery. Reads from the *B. hookeriana* samples were mapped onto the reference using SOAPsnp (with parameters -u t -Q i -L 90) to detect the SNP [47]. SOAPsnp calculates the likelihood of each genotype at each site and then infers the genotype using highest posterior probability at each site based on Bayes’ theorem following a reverse probability model [47]. The SNP sites were filtered according to different conditions: (1) all samples have the same type of SNP on a certain site; (2) all samples have an SNP on a certain site, but the types are not all the same; (3) all samples have coverage on a certain site, and at least one of the samples has an SNP; (4) all samples have coverage on a certain site, and at least two different types of SNPs appear in the samples on a certain site, and (5) all sites. To determine the homozygosity within a genotype, a base needs to be supported by at least 80% of the reads.

### Comparative analysis of *B. hookeriana*, *N. nucifera*, and *A. thaliana* transcriptomes

Transcriptome of *B. hookeriana* was further compared with that of *N. nucifera* and *A. thaliana*. First, we downloaded the assembled transcriptomes from *N. nucifera* leaves [16], and leaf cDNA data of *A. thaliana* [17]. Both transcriptomes were aligned with the *B. hookeriana* transcriptome using TBLASTX with E-value cut-off of 1E–10. If the transcripts of *N. nucifera/A. thaliana* only mapped with a single *B. hookeriana* unigene, it was considered to have a one-to-one relationship. Both leaf transcriptomes were annotated with Swiss-Prot and KEGG database with the same settings as described. GO annotations were obtained using BLAST2GO from the Swiss-Prot annotated transcripts [48]. We then used WEGO for GO functional classification of transcripts [49]. All annotated transcripts were mapped to GO terms, and then the number of transcripts associated with each term was calculated. The KEGG pathway annotations of *B. hookeriana*, *N. nucifera*, and *A. thaliana* were performed using KOBAS 2.0 [50].

### SSR development and primer design

We implemented SSR analyses using MicroSAtellite identification tool (MISA) (http://pgrc.ipk-gatersleben.de/misa) to identify perfect di-, tri-, tetra-, penta-, and hexa-nucleotide with minimum repeats of 6, 5, 5, 4, and 4, respectively. The SSRs with >150 bp flanking regions on the unigenes were kept for primer design purpose. The SSR primer design was done using Primer3 [51]. We set the following parameters for primer design: (1) three mismatches were allowed for the primers aligned to the unigene 5′ site and only one mismatch was allowed in the 3′ site; and (2) the primers can only be aligned to a single unigene. SSR Finder was used to search SSRs on *B. hookeriana* unigenes [52]. If the SSR found in SSR Finder matched with SSR from MISA, the products were kept as potential SSR markers.

## Authors’ contributions

TH and NJE conceived the project and designed the experiments; HMD performed glasshouse experiment; SLL performed interpretation of gene expression data and bioinformatics, annotated data, and prepared the figures and drafted the manuscript. All authors were involved in the manuscript revision, read and approved the final manuscript.

## Competing interests

The authors declare no competing financial interests.

## Figures and Tables

**Table 1:** The initial sequencing output statistics in four *B. hookeriana* leaf samples

Sample	Total No. of raw reads	Total No. of clean reads	Total No. of clean bases	Base call accuracy (%)	GC content (%)
A	50,007,092	46,429,128	4,642,912,800	97.90	45.53
B	49,999,398	46,813,630	4,681,363,000	97.90	45.66
C	50,696,470	47,472,990	4,747,299,000	97.88	45.88
D	51,769,470	48,432,518	4,843,251,800	97.88	45.84
Mean	50,618,108	47,287,067	4,728,706,650	97.89	45.73

**Table 2:** Contig and unigene assembly of *B. hookeriana* leaf transcriptome

Assembly	Sample	Total No. of contigs/unigenes	Total length (nt)	Mean length (nt)	N50	Total No. of consensus sequences	Total No. of distinct clusters	Total No. of distinct singletons
Contigs	A	93,281	37,550,807	400	880	–	–	–
	B	104,947	41,313,733	394	869	–	–	–
	C	98,063	40,097,562	409	925	–	–	–
	D	100,923	40,874,608	405	909	–	–	–
	Mean	99,304	39,959,178	402	896	–	–	–
Unigenes	A	53,873	40,258,014	747	1440	53,873	17,236	36,637
	B	59,340	46,401,909	782	1528	59,340	19,229	40,111
	C	55,176	43,281,707	784	1504	55,176	18,388	36,788
	D	56,904	44,344,372	779	1507	56.904	18,814	38,090
	Overall	59,063	64,827,597	1098	1813	59,063	25,912	33,151

*Note*: Overall values were calculated based on the entire library.

**Table 3:** SNP discovery from *B. hookeriana* leaf transcriptome

Sample	Total No. of SNPs	No. of SNPs per 1 kb	Heterozygosity	Transition	Transversion	No. of unigenes
A	39,485	0.609	28,618	25,069	14,416	13,818
B	36,611	0.565	16,424	23,287	13,324	13,122
C	44,330	0.684	31,948	28,224	16,106	15,255
D	46,170	0.712	33,373	29,363	14,416	13,818

*Note*: SNP, single nucleotide polymorphism.

**Table 4:** Top five KEGG pathways enriched in *B. hookeriana*

Pathway name	Pathway ID	Genes contained, number (%)
Metabolic pathways	ko01100	3704 (13.86%)
Biosynthesis of secondary metabolites	ko01110	2235 (8.36%)
Plant–pathogen interaction	ko04626	1418 (5.31%)
Plant hormone signal transduction	ko04075	1039 (3.88%)
Spliceosome	ko03040	666 (2.49%)

## Supplementary material

Supplementary Table 1 The *B. hookeriana* unigenes matching with a protein in Swiss-Prot database in 80%–100% of its length

Supplementary Table 2 The third-level GO term enrichment of *B. hookeriana*, *N. nucifera*, and *A. thaliana*

Supplementary Table 3 Potential *B. hookeriana* SSR markers associated with the predicted primers
